# Antiretroviral Treatment and Prevention of Peripartum and Postnatal HIV Transmission in West Africa: Evaluation of a Two-Tiered Approach

**DOI:** 10.1371/journal.pmed.0040257

**Published:** 2007-08-21

**Authors:** Besigin Tonwe-Gold, Didier K Ekouevi, Ida Viho, Clarisse Amani-Bosse, Siaka Toure, Patrick A Coffie, François Rouet, Renaud Becquet, Valériane Leroy, Wafaa M El-Sadr, Elaine J Abrams, François Dabis

**Affiliations:** 1 MTCT-Plus Programme, ACONDA, Abidjan, Côte d'Ivoire; 2 Institut de Santé Publique, Epidémiologie et Développement, Université Victor Segalen, Bordeaux, France; 3 INSERM Unité 593, Bordeaux, France; 4 ANRS 1201/1202, Ditrame Plus Project, PACCI Collaboration, Abidjan, Côte d'Ivoire; 5 CeDReS Laboratory, Centre Hospitalier Universitaire Treichville, Abidjan, Côte d'Ivoire; 6 MTCT-Plus Initiative, International Center for AIDS Care and Treatment Programs, Mailman School of Public Health, Columbia University, New York, New York, United States of America; National Institute of Child Health and Human Development, United States of America

## Abstract

**Background:**

Highly active antiretroviral treatment (HAART) has only been recently recommended for HIV-infected pregnant women requiring treatment for their own health in resource-limited settings. However, there are few documented experiences from African countries. We evaluated the short-term (4 wk) and long-term (12 mo) effectiveness of a two-tiered strategy of prevention of mother-to-child transmission of HIV (PMTCT) in Africa: women meeting the eligibility criteria of the World Health Organization (WHO) received HAART, and women with less advanced HIV disease received short-course antiretroviral (scARV) PMTCT regimens.

**Methods and Findings:**

The MTCT-Plus Initiative is a multi-country, family-centred HIV care and treatment program for pregnant and postpartum women and their families. Pregnant women enrolled in Abidjan, Côte d'Ivoire received either HAART for their own health or short-course antiretroviral (scARV) PMTCT regimens according to their clinical and immunological status. Plasma HIV-RNA viral load (VL) was measured to diagnose peripartum infection when infants were 4 wk of age, and HIV final status was documented either by rapid antibody testing when infants were aged ≥ 12 mo or by plasma VL earlier. The Kaplan-Meier method was used to estimate the rate of HIV transmission and HIV-free survival. Between August 2003 and June 2005, 107 women began HAART at a median of 30 wk of gestation, 102 of them with zidovudine (ZDV), lamivudine (3TC), and nevirapine (NVP) and they continued treatment postpartum; 143 other women received scARV for PMTCT, 103 of them with sc(ZDV+3TC) with single-dose NVP during labour. Most (75%) of the infants were breast-fed for a median of 5 mo. Overall, the rate of peripartum HIV transmission was 2.2% (95% confidence interval [CI] 0.3%–4.2%) and the cumulative rate at 12 mo was 5.7% (95% CI 2.5%–9.0%). The overall probability of infant death or infection with HIV was 4.3% (95% CI 1.7%–7.0%) at age week 4 wk and 11.7% (95% CI 7.5%–15.9%) at 12 mo.

**Conclusions:**

This two-tiered strategy appears to be safe and highly effective for short- and long-term PMTCT in resource-constrained settings. These results indicate a further benefit of access to HAART for pregnant women who need treatment for their own health.

## Abstract 2

In an observational cohort study from Côte d'Ivoire, François Dabis and colleagues report on prevention of mother-to-child HIV transmission among women receiving antiretroviral therapy according to World Health Organization recommendations.

## Editors' Summary

**Background:**

Effective treatments are available to prevent AIDS in people who are infected with HIV, but not everyone with HIV needs to take medication. Usually, anti-HIV medication is recommended only for those whose immune systems have been significantly affected by the virus, as evidenced by symptoms or by the results of a blood test, the CD4 lymphocyte (“T cell”) count. Treating HIV usually requires a combination of three or more medications. These combinations (called HAART) must be taken every day, can cause complications, and can be expensive.

Worldwide, more than half a million children became infected with HIV each year. Most of these children acquire HIV from their mothers during pregnancy or around the time of birth. If a pregnant woman with HIV takes HAART, her chances of passing HIV to the baby are greatly reduced, but the possible side effects of HAART on the baby are not known. Also, most transmission of HIV from mothers to babies occurs in poor countries where supplies of HAART are limited. For these reasons, World Health Organization (WHO) does not recommend that every pregnant woman receive HAART to prevent HIV transmission to the baby, unless the woman needs HAART for her own health (for example if her T cells are low or she has severe symptoms of HIV infection). For pregnant women with HIV who do not need to take HAART for their own health, less complicated treatments, involving a short course of one or two HIV drugs, can be used to reduce the risk of passing HIV to the baby.

**Why Was This Study Done?:**

The WHO recommendations for HAART in pregnancy are based on the best available evidence, but it is important to know how well they work in actual practice. The authors of this study were providing HIV treatment to pregnant women with HIV in West Africa through an established clinic program in Abidjan, Côte d'Ivoire, and wanted to see how well the WHO recommendations for HAART or short-course treatments, depending on the mother's condition, were working to protect babies from HIV infection.

**What Did the Researchers Do and Find?:**

The researchers studied 250 HIV-infected pregnant women who received HIV medications in the Abidjan program between mid-2003 and mid-2005. In accordance with WHO guidelines, 107 women began HAART for their own health during pregnancy, and 143 women did not qualify for HAART but received other short course treatments (scARV) to prevent HIV transmission to their babies. The authors monitored mothers and babies for treatment side effects and tested the babies for HIV infection up to age 1 y.

They found that HAART was relatively safe during pregnancy, although babies born to women on HAART were more likely (26.3%) to have low birth weight than babies born to women who received scARV (12.4%). Also, 7.5% of women on HAART developed side effects requiring a change in their medications. Combining the results from HAART and scART groups, the chance of HIV transmission around the time of birth was 2.2%, increasing to 5.7% at age 1 y. (Three-quarters of the infants were breast-fed; safe water for mixing formula was not reliably available.) The study found no difference in risk of HIV infection between babies whose mothers received HAART and those whose mothers received scARV according to guidelines.

**What Do These Findings Mean?:**

These results support the safety and effectiveness of the WHO two-tiered approach for preventing mother-to-child transmission. This study was not designed to compare HAART to scART directly, because the women who received HAART were the ones with more advanced HIV infection, which might have affected their babies in many ways.

Compared to earlier pregnancy studies of HAART in rich countries, this study of the WHO approach in West Africa showed similar success in protecting infants from HIV infection around the time of birth. Unfortunately, because formula feeding was not generally available in resource-limited settings, protection declined over the first year of life with breast-feeding, but some protection remained.

This study confirms that close monitoring of pregnant women on HAART is necessary, so that drugs can be changed if side effects develop. The study does not tell us whether using scARV in pregnancy might change the virus in ways that would make it more difficult to treat the same women with HAART later if they needed it. The reason for low birth weight in some babies born to mothers on HAART is unclear. It may be because the women who needed HAART had more severe health problems from their HIV, or it may be a result of the HAART itself.

**Additional Information.:**

Please access these Web sites via the online version of this summary at http://dx.doi.org/10.1371/journal.pmed.0040257.

- World Health Organization has a page on prevention of mother-to-child transmission of HIV
- “Women, Children, and HIV” is a resource site from the François Xavier Bagnoud Center and UCSF
- The MTCT-Plus initiative at Columbia University supports the programs in Abidjan

## Introduction

Mother-to-child transmission (MTCT) of HIV-1 is estimated to be the cause of at least 90% of paediatric HIV infections, with more than 700,000 children newly infected in 2006 worldwide [1]. New HIV infections in children are becoming increasingly rare in Western Europe (200 reported in 2004) and the United States (300) [1,2]. In these relatively resource-rich contexts, the availability of highly active antiretroviral therapy (HAART), usually three antiretroviral (ARV) drugs during pregnancy, in combination with the avoidance of breast-feeding and with elective caesarean section, has reduced the transmission rate to less than 2% and resulted in the near elimination of MTCT [1–3].

In sub-Saharan Africa, progress has been made in the last few years toward expanding prevention of MTCT (PMTCT) programs based on trial findings that have demonstrated the efficacy of generally simpler and shorter ARV prophylactic regimens than those used in industrialized countries [4–9]. This expansion has been based primarily on the use of single-dose nevirapine (sdNVP) and, to a lesser extent, short-course antiretroviral (scARV) regimens of one or two drugs administered at the end of pregnancy and followed with sdNVP to prevent peripartum transmission. Postnatal transmission of HIV, resulting in 9.8 new HIV infections per 100 child-years of breast-feeding overall, and occurring most frequently in women with low CD4 T cell counts [10], reduces the long-term overall efficacy of peripartum ARV regimens [11].

World Health Organization (WHO) had set a target of treating three million individuals in the developing world with HAART by 2005 [12], and the United Nations General Assembly Special Session on HIV/AIDS committed countries to reducing by 20% the proportion of infants infected with HIV by 2005 [13]. In this context, WHO guidelines for the prevention of perinatal transmission in low-resource settings now recommend using HAART for pregnant women in need of ARV therapy (ART) for their own health [14]. This policy was agreed upon in recognition of the potential impact of HAART on decreasing morbidity and mortality in women with advanced HIV disease, as well as its potentially profound effect on preventing peripartum and postnatal HIV infection. Currently, access to HAART for pregnant women in low-income countries is limited, and there are few documented experiences from African countries [15]. Although HAART is widely used for peripartum PMTCT in industrialized countries [2,3,16], its efficacy and safety have not been fully assessed in resource-limited settings characterised by high HIV seroprevalence, and few data are available to assess the impact of ART on postnatal transmission.

We evaluated a two-tiered PMTCT strategy in pregnant women enrolled at the MTCT-Plus Initiative care and treatment program in Abidjan, Côte d'Ivoire: on the basis of their medical status (CD4 T cell count and WHO stage) they were given either HAART or scARV for PMTCT. HAART was targeted at pregnant women with advanced HIV disease who met WHO eligibility criteria for treatment and scARV regimens for PMTCT were prescribed to women with less advanced HIV disease. We report the safety of HAART in pregnant women and the 4 wk and 12 mo outcomes for infant HIV infection and mortality for this stepped PMTCT strategy.

## Methods

### Study Design and Participants

This observational cohort included women enrolled in the MTCT-Plus Initiative, a multi-country, comprehensive HIV care and treatment program for pregnant and postpartum women and their families built on existing PMTCT services. It provides pregnant and postpartum women with holistic, family-centred HIV care including HAART to the woman, her partner, and her children [17]. Pregnant women identified as HIV-infected at two community-based antenatal clinics in two low-income urban districts of Abidjan were referred for enrolment into the MTCT-Plus program. The study population included all HIV-infected pregnant women and their live-born infants enrolled between August 2003 and April 2005 and followed until a final paediatric HIV status could be determined. Women were divided into two groups. The first cohort included the HIV-infected pregnant women who were eligible for HAART based on WHO criteria (at high risk of transmission); the second cohort included HIV-infected pregnant women who were not eligible for HAART and received sc-PMTCT (with low risk of transmission).

The MTCT-Plus program was reviewed by the institutional review board (IRB) from Columbia University in 2000 (principal sponsor) and was not considered a research project but rather a demonstration program in the context of the ARV roll-out. Informed consent was not required by the Columbia IRB.

### Antiretroviral Regimens

Upon enrolment, all participants were screened for HAART eligibility, defined from August 2003 to December 2004 as: WHO clinical stage 4 irrespective of CD4 T cell count; WHO stage 2 or 3 and CD4 T cell count ≤ 350 cells/mm^3^; or CD4 T cell count < 200 cells/mm^3^. These criteria were revised by the MTCT-Plus Initiative in January 2005 to be in compliance with WHO 2005 PMTCT guidelines and to avoid the side effects reported with NVP especially in pregnant women with CD4 T cell count > 250 cells/mm^3^ [18]; from that time forward, women with WHO stage 2 and CD4 T cell count < 350 cells/mm^3^ were no longer considered eligible for HAART*.*

Other criteria taken into account for deciding to initiate HAART were the absence of medical contraindications to HAART as well as major barriers to adherence. Pregnant women who met eligibility criteria initiated HAART as early as 24 wk of gestation according to the last menstruation date, usually with zidovudine (ZDV), lamivudine (3TC) and nevirapine (NVP). Treatment continued during labour and postnatally. Pregnant women who were not eligible for HAART received validated scARV prophylactic regimens, usually sc(ZDV+3TC) from 32 wk of gestation (until 3 d postpartum) and sdNVP in labour [9,19], or scZDV from 28 wk, or sdNVP alone, or both scZDV and sdNVP [4,7–9,20]. All infants received ZDV syrup for 7 d and sdNVP syrup on day 3 irrespective of the maternal drug regimen.

### Follow-Up Procedures

Clinical classification using the WHO staging system was performed by the physicians at enrolment into the MTCT-Plus program and at each subsequent visit.

Women either used breast milk substitutes if feasible and affordable following WHO guidelines [21] or were encouraged to practice exclusive breast-feeding for a maximum of 6 mo and initiate early weaning from 4 mo onwards. Caesarean section was only performed for emergencies. Baseline adherence and psycho-social assessments were done before initiation of HAART, and followed by weekly visits for 8 wk using a standardized checklist of clinical symptoms to detect side effects and ensure adherence. A more detailed clinical evaluation, including symptom review, physical examination, and review of medication adherence was conducted monthly following MTCT-Plus clinical guidelines [22]. Adherence was assessed by physicians or nurses on the basis of a self report consisting of the number of pills taken by the women during the last 7 d preceding each schedule visit (all, most, few, none). Severe adverse events were classified according to international guidelines [23].

### Laboratory Procedures

All blood samples of pregnant women who agreed to test were screened on site for HIV antibodies according to a validated algorithm described elsewhere [24]. CD4 T cell count was measured upon enrolment and at intervals of 6 mo. Hepatic and renal functions were measured prior to HAART initiation; liver function tests were repeated 2 wk after treatment initiation and monthly throughout pregnancy for women on HAART. Additional laboratory evaluation was restricted to patients with clinical indications.

Laboratory tests were done at a central reference laboratory (CeDReS, Abidjan) certified by regional and international quality assurance programs.

The CD4 T cell count and percentage were measured in the antenatal period at the enrolment visit and thereafter every 6 mo after the initiation of the treatment using a dual-platform flow-cytometry technique with an automated blood cell counter (MaxM, Beckman Coulter, http://www.beckmancoulter.com/) for absolute lymphocyte count and a flow cytometer (FACScan, Becton Dickinson, http://www.bdbiosciences.com/) for measuring the percentage of CD4 T cells (CD4%). Absolute CD4 T cell count was then calculated multiplying the CD4% by the total lymphocyte count. The laboratory quality control procedures included: (i) daily internal controls for both the automated blood cell counter (Coulter 5C cell control, Beckman Coulter) and the flow cytometer (BD multi-check control, Becton Dickinson); (ii) participation in two international assurance quality programs (UK-NEKAS and QASI).

Plasma HIV-RNA viral load (VL) testing for the early diagnosis of paediatric HIV infection was performed using a quantitative real-time RT-PCR technique targeted at the HIV-1 LTR gene as previously validated [25]. The quantification limit of this method was 300 copies/ml with 200 μl of plasma. All infants were tested at 4 wk of age, and if positive, confirmed with repeat VL testing at 6 wk. Infants with two positive tests were classified as infected peripartum. Infants with a negative VL test at 4 wk of age were classified as uninfected peripartum and were subsequently tested at 12 mo by rapid HIV antibody testing. If the antibody test at 12 mo was positive, we performed a second test for confirmation at 15 mo or later. The HIV infection status of the children who stopped follow-up just after being tested HIV antibody-positive at 12 mo or who had ceased breast-feeding under the age of 2 mo was confirmed by VL testing. HIV antibody-negative or VL-negative children were classified as uninfected. Postnatal transmission was defined as a negative HIV PCR from a first sample at age 30–180 d followed by a positive VL or antibody test at 12 or 15 mo.

### Statistical Analysis

Group comparisons according to ARV regimens used Student *t*-test or nonparametric Mann-Whitney test for quantitative variables, and Chi^2^ test or Fisher exact test for qualitative variables. The first-live born child was used for analysis in the case of multiple births.

Two survival analyses were conducted using two different outcomes in children: HIV infection or HIV-free survival (which is defined as ending at the occurrence of HIV infection or death, whichever came first). Cumulative transmission risks of HIV and (HIV or death) were estimated using both Turnbull method and Kaplan-Meier probabilities. As results were similar (unpublished data), Kaplan-Meier estimates were retained to allow for comparisons between groups using the log-rank test. For survival analysis, time to infection was estimated up to the first positive test for peripartum cases or to the midpoint date between the last negative test and the first positive test for the postnatal cases. Results were expressed in percentages with their 95% confidence intervals (95% CIs). Determinants of HIV infection and (HIV or death) were explored using a Cox model with the following variables: maternal age, ARV regimen, WHO staging, CD4 T cell count, haemoglobinemia, infant feeding practice from birth, birth weight, and child gender. A stepwise descendant multivariate analysis included all variables with *p* < 0.25 in the univariate analysis. Statistical analyses were processed with Stata software, version 9.0 (Stata, http://www.stata.com/).

## Results

### Characteristics of Study Population

**Figure 1:**
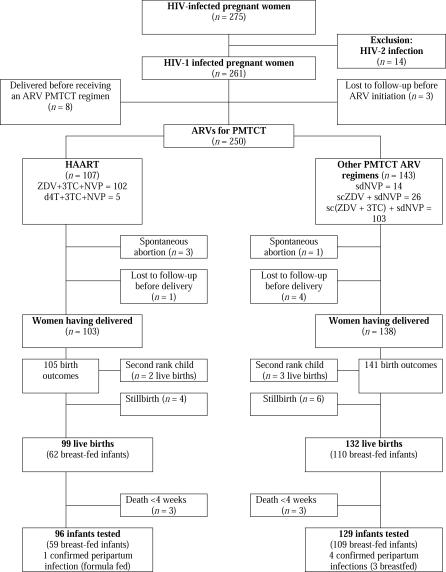
Study Profile Population included in the estimation of the rate of mother-to-child transmission of HIV in the MTCT-Plus program in Abidjan, Côte d'Ivoire, August 2003 through October 2006. Among the six HIV-infected infants, three had received single dose nevirapine (sdNVP), two short courses of ZDV+sdNVP, and one short course of ZDV+3TC+sdNVP for PMTCT.

Between August 2003 and April 2005, 261 pregnant women with confirmed HIV-1 infection were enrolled and 250 were prescribed ARVs before and/or during delivery: 143 (57%) received scARVs and 107 (43%) received a HAART regimen. Three of the original 261 women were lost to follow-up and eight delivered before an ARV regimen could be initiated (Figure 1).

Maternal baseline characteristics are summarized in Table 1. Overall, median maternal age was 27 y (interquartile range [IQR] 24–31 y) and median gestational age at the time of enrolment was 30 wk (IQR 25–33 wk). Median CD4 T cell count was 338 cells/mm^3^ (IQR 206–488 cells/mm^3^) and 44 (17.6%) of the women were classified as WHO clinical stage 3 or 4.

**Table 1:**
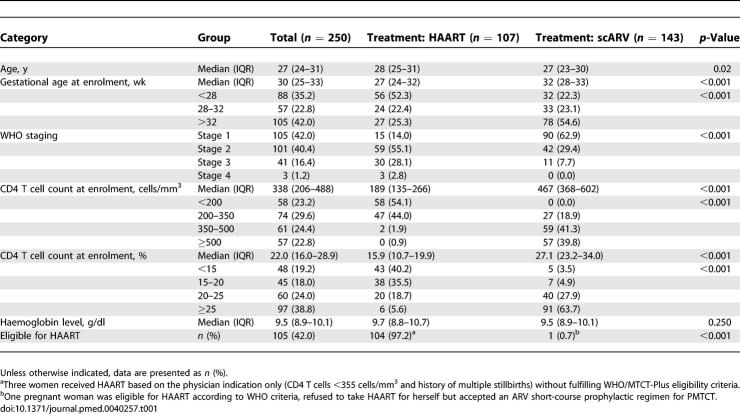
Maternal Characteristics of HIV-Infected Pregnant Women Having Initiated an ARV Regimen for PMTCT in the MTCT-Plus Program, Abidjan (Côte d'Ivoire), 2003–2005

HAART eligibility criteria were met by 104 women; in addition, three women received HAART at the discretion of the physician without fulfilling WHO/MTCT-Plus criteria. The median CD4 T cell count of all 107 women who received HAART was 189 cells/mm^3^ (IQR 135–266 cells/mm^3^); 33 (30.9%) of these women were at WHO stage 3 or 4, and 58 (54.1%) had a CD4 T cell count < 200 cells/mm^3^ (Table 1). Most (102 [95.3%]) received a combination of ZDV, 3TC, and NVP. Five women (4.7%) were prescribed stavudine (d4T), 3TC, and NVP (Figure 1). HAART was initiated at a median of 30 wk of gestation (IQR 27–33 wk) (Table 2). Median duration on HAART until delivery was 74 d (IQR 48–91 d). Of the 93 women treated with HAART ≥ 1 mo before delivery, 91% reported complete adherence. All infants born to HAART-treated women received sdNVP at 3 d of life and 1 wk of ZDV syrup.

**Table 2:**
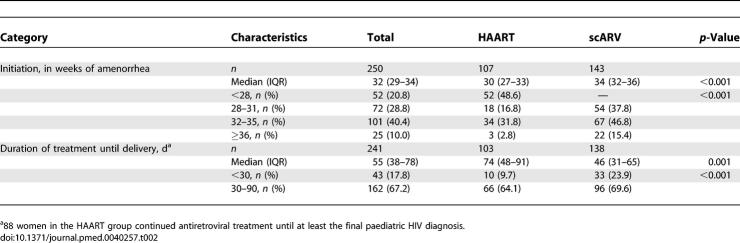
Antiretroviral Drug Regimen Characteristics in Pregnant Women Enrolled in the MTCT-Plus Program in Abidjan (Côte d'Ivoire), 2003–2005

Among the 143 pregnant women who received scARV for PMTCT, the median CD4 T cell count was 467 cells/mm^3^ (IQR 368–602 cells/mm^3^) and 11 (7.7%) had WHO stage 3 HIV disease at the time of enrolment (Table 1). Median gestational age at prophylaxis initiation was 34 wk (IQR 32–36 wk) and median number of days on scARV drug regimens was 46 d (IQR 31–65 d) (Table 2); 103 women (72.0%) received sc(ZDV+3TC) with sdNVP during labour, 26 (18.2%) scZDV with sdNVP, and 14 (9.8%) sdNVP alone (Figure 1).

Among the 241 women who had pregnancy outcomes for the analysis (Figure 1), 172 (71.3%) initiated breast-feeding at birth (62 in the HAART group and 110 in the scARV for PMTCT group). The overall median duration of breast-feeding was 5.4 mo (IQR 4.0–6.8 mo) and was slightly longer in the scARV for PMTCT group (5.8 mo) than in the HAART group (4.6 mo) (*p* = 0.010).

### Effectiveness of ARV Regimens at 4 Weeks and 12 Months

**Figure 2:**
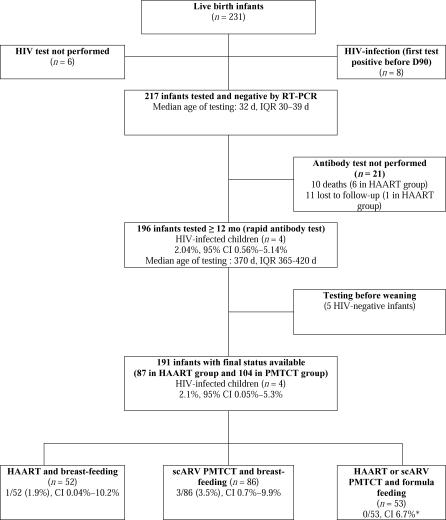
Postnatal HIV-1 Transmission Profile of postnatal transmission study in the MTCT-Plus Program in Abidjan, Côte d'Ivoire, August 2003 through October 2006 *Upper limit of 95% confidence interval.

Excluding the second child of five multiple births, there were 241 pregnancy outcomes for the present analysis (Figure 1). Four (3.9%) and 6 (4.3%) stillbirths were reported in the HAART and the scARV for PMTCT groups, respectively (*p* = 1.00). HIV infection status could not be determined for six live-born children (three in each group), who died in the first week of life before a blood sample could be collected (Figure 1). Overall, among the 231 neonates at risk of HIV infection, 172 (75.1%) initiated breast-feeding, of whom 168 were tested for HIV at the age of 4 wk. Overall HIV infection status could be ascertained for 225 children (97.4%), among whom 12 were diagnosed as HIV-infected (Table 3): five were diagnosed by the age of 4 wk, three more by the age of 3 mo, and the remaining four by the age of 12 mo (Figure 2). The overall probability of confirmed peripartum HIV infection was 2.2% (95% CI 0.3%–4.2%): 1.0% (95% CI 0.0%–3.1%) in the HAART group and 3.1% (95% CI 0.1%–6.1%) in the scARV for PMTCT group (Table 4). At 12 mo, 5.7% (95% CI 2.5%–9.0%) of the infants were diagnosed as HIV-infected: 3.3% (95% CI 0.0%–6.9%) in the HAART group and 7.5% (95% CI 2.8%–12.3%) in the scARV for PMTCT group (log-rank test, *p* = 0.18). No statistically significant difference was found according to the infant feeding practice (*p* = 0.48). In multivariate analysis, low birth weight (<2,500 g) was the only factor associated with acquisition of HIV infection, with an adjusted hazard ratio of 5.63 (95% CI 1.62–19.49, *p* = 0.006), controlling for infant feeding practice, gender, maternal ARV drug regimen, WHO staging, absolute CD4 T count, haemoglobin level, and age at baseline.

**Table 3:**
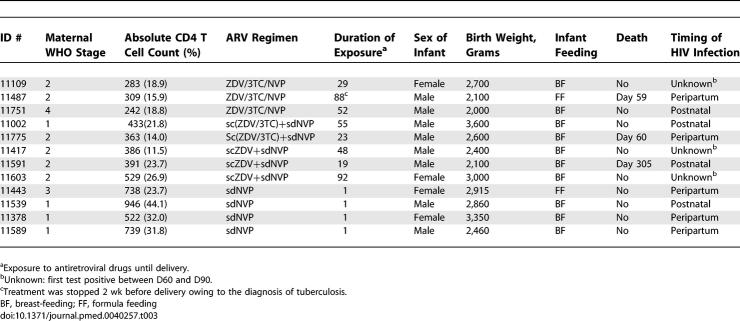
Description of the 12 Cases of Paediatric HIV Infection in the MTCT-Plus Program in Abidjan (Côte d'Ivoire), 2003–2006

**Table 4:**
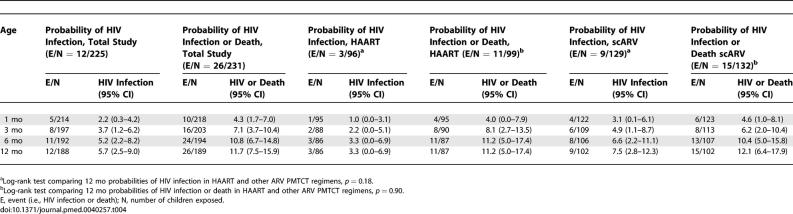
Kaplan-Meier Probabilities of HIV Infection in Children Born Alive and Probabilities of Acquiring HIV or Dying MTCT-Plus Program, Abidjan (Côte d'Ivoire), 2003–2006

### Postnatal Transmission

The postnatal transmission risk was estimated among all children who were RT-PCR HIV-negative at age 4 wk (Figure 2). Subsequent pediatric diagnostic assessment was not possible for 21 children: 11 were lost to follow-up (ten in the scARV for PMTCT group and one in the HAART group), and ten had died (four in the scARV for PMTCT group and six in the HAART group). The definitive HIV infection status could not be determined for five children who were antibody-negative but still breast-fed at 12 mo of age. A total of 191 infants were thus included in this analysis (87 in the HAART group and 104 in the scARV for PMTCT group) including 138 breast-fed infants (52 in the HAART group and 86 in the scARV for PMTCT group). The median duration on HAART in breast-feeding women was 14.9 mo (IQR 14.5–16.2 mo) when the final paediatric HIV status was ascertained. Overall, four infants had a confirmed postnatal infection (2.3% [95% CI 0.8%–7.3%]). In the HAART group, one case of postnatal infection was identified among 52 infants (1.9% [95% CI 0.04%–10.2%]) breast-fed for a median of 4.7 mo (IQR 3.3–6.3 mo). In the scARV for PMTCT group, three cases of postnatal infection were identified among 86 infants (3.5% [95% CI 0.7%–9.9%]) breast-fed for a median of 5.7 mo (IQR 4.3–7.1 mo). If we hypothesize that the three cases with unknown timing are possible postnatal cases, the rate of postnatal transmission could be 3.8% (0.4%–12.9%) in the HAART group and 5.7% (1.8%–12.7%) in the PMTCT group. No case of postnatal infection was identified among the 53 infants who were formula fed (upper limit of 95% CI, 6.7%). One death of an HIV-infected formula-fed infant was reported (Table 5).

**Table 5:**
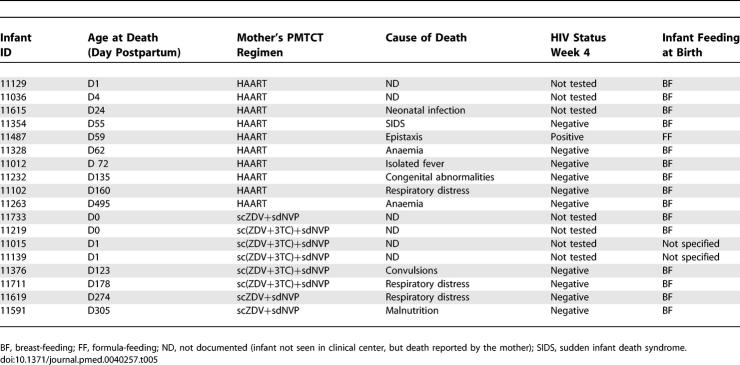
Description of the Occurrence of Pediatric Death in the MTCT-Plus Program in MTCT-Plus Program, Abidjan (Côte d'Ivoire), 2003–2006

### 12-Month Infant HIV-Free Survival and Death According to Maternal ARV Regimens

The overall probability of HIV infection or death among infants aged 12 mo of all women enrolled in this study was 11.7% (95% CI 7.5%–15.9%); the probability was 11.2% (95% CI 5.0%–17.4%) among infants born to HAART-treated women, and 12.1% (95% CI 6.4%–17.9%) among infants whose mothers received scARV for PMTCT only (log-rank test, *p* = 0.90) (Table 4). In multivariate analysis, two factors were associated with the occurrence of HIV infection or death (*n* = 231): low birth weight (adjusted hazard ratio = 3.71 [95% CI 1.53–9.03], *p* = 0.004) and female sex (adjusted hazard ratio = 0.35 [95% CI 0.14–0.88], *p* = 0.035), after controlling for infant feeding practice, maternal ARV drug regimen, WHO staging, absolute CD4 T count, haemoglobin level, and age at baseline.

A total of 18 deaths were reported in this cohort: of 99 infants born to woman who had initiated HAART during pregnancy, ten (10.1%) died, and among 132 infants who were born to women who received short-course antiretroviral prophylaxis, eight (6.0%) died. The data on infant mortality are summarized according to the maternal ARV regimen in Table 5. Twelve of the 18 deaths occurred in a medical facility where a plausible cause was recorded. Due to the intensive follow-up of mothers on HAART, eight out of the ten infant deaths in this group were documented, compared to only four of the eight deaths in the scARV for PMTCT group.

### Maternal Adverse Events

As detailed in Table 6, nine (8.4%) of 107 pregnant women initiating HAART required one change to their ARV regimen before delivery. One woman was changed from NVP to efavirenz at week 6 of treatment (gestational age 32 wk) after diagnosis of tuberculosis. Eight women (7.5%) developed grade 3 or 4 adverse events attributed to HAART: five women developed grade 3 mucocutaneous rash attributed to NVP and were switched to nelfinavir, and two women developed grade 4 liver toxicity attributed to NVP, with alanine aminotransferase levels over 50 times and over 5 times the upper limit of normal value, respectively in the two groups of women; one woman developed severe anaemia attributed to ZDV. All events resolved with drug changes and no deaths were reported. In the cohort of women receiving scARV for PMTCT, no grade 3 or 4 toxicities were observed and no ART changes were made.

**Table 6:**
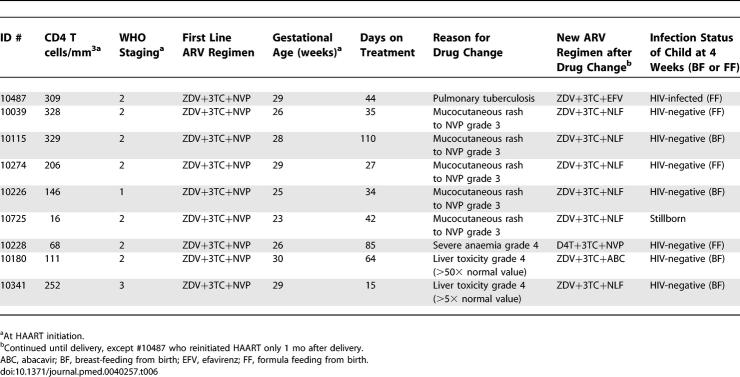
Pregnant Women Initiating HAART and Requiring ARV Drug Change before Delivery in the MTCT-Plus Program in Abidjan, Côte d'Ivoire, 2003–2006

### Neonatal Adverse Events

As shown in Figure 1, the frequency of perinatal deaths (stillbirths and neonatal deaths in the first 28 d of life) was comparable in the HAART and scARV for PMTCT groups: 6.7% and 5.6%, respectively (*p* = 0.80). No significant difference in the rates of premature births (gestational age <37 wk) was noted between the two groups (7.8% and 7.9% respectively, *p* = 1.00). However, a higher proportion of low birth weight (<2,500 g) was observed in neonates whose mothers had received HAART, 26.3% compared with 12.4% among those having received a scARV for PMTCT regimen (*p* < 0.001). These differences persisted after adjusting for maternal body mass index, CD4 T cell count, and WHO clinical stage.

## Discussion

We evaluated a two-tiered PMTCT strategy in which treatment was selected based on maternal medical status (as indicated by CD4 T cell count and WHO staging) in a West African population. HAART was prescribed to pregnant women with advanced HIV disease who met WHO eligibility criteria based on their own health status, whereas short-course various combinations of ARV regimens for PMTCT were given to pregnant women with less advanced HIV disease, who did not qualify for HAART. Three-quarters of the infants were breast-fed, for a median of 5.4 mo. Overall, the rate of peripartum HIV transmission was 2.2%, the cumulative rate of infant HIV infection at 12 mo was 5.7%, and the 12-mo HIV-free survival was 88.3%, without measurable differences between the two groups.

The rate of peripartum MTCT that we observed in women on HAART was very low (1.0%), similar to rates reported from industrialized countries [2,3], despite a later identification of HIV infection during pregnancy, and a relatively short duration of HAART prior to delivery. In the Women and Infants Transmission Study in the US, HIV-1 transmission rate was 1.2% in infants whose mothers had received HAART but did not breast-feed [3]. Similarly, the European Collaborative Study Group reported the decline of MTCT transmission rates from 15.5% in 1994, to 5.1% in 1997–1998, and down to 0.99% in 2001–2002, i.e., when HAART became more widely used [2].

The maternal and PMTCT benefits of HAART initiated during pregnancy that have been described in the industrialized world may differ for populations from sub-Saharan Africa because of the wider spectrum of opportunistic diseases, the deeper level of immunodeficiency at which they occur [26], and the poorer nutritional background and obstetrical conditions. In the DREAM cohort in Mozambique where formula feeding was systematic, pregnant women initiated a ZDV/NVP/3TC HAART combination at 25 wk of gestation, irrespective of clinical or immunological staging; the rate of HIV transmission from mother to infant was 1.2% at infant age of 1 mo [27]. HAART was continued in this cohort up to 6 mo postdelivery for those not requiring therapy for their own health, while the others continued treatment indefinitely. We observed a comparable 1 mo of age rate of transmission with a more targeted prescription of HAART. As emphasized in the recently published WHO PMTCT guidelines, it is critically important that pregnant women who are eligible for HAART receive such treatment for the sake of their own health as well as for reducing MTCT [14]. Women with advanced HIV disease are at the highest risks both for HIV-related morbidity and mortality and for transmitting HIV to their child [28]. By decreasing the mother's plasma HIV VL and enhancing immune function, a targeted HAART both treats the mother's own HIV infection and reduces dramatically the likelihood of HIV transmission to the newborn. If HAART is used for PMTCT in women not requiring it for their own health, however, the potential risk to the woman's health of interrupting HAART after delivery and the potential risks of continuous HAART exposure to uninfected infants are unclear.

We observed low rates of postnatal transmission in both HAART-treated women and those receiving scARV for PMTCT only. These results were observed in conditions of short breast-feeding exposure with an overall median duration of 5.4 mo. We observed one case of postnatal HIV transmission among 52 breast-fed infants of HAART-treated mothers and 3/86 among the breast-fed infants of women on scARV regimens. There was no statistically significant difference between the two groups, although sample size was limited; this study was not designed to answer the question “does HAART substantially reduce postnatal transmission risk? While HAART might diminish postnatal MTCT because it suppresses cell-free HIV RNA in breast milk, other data counteract this hypothesis [29]. Further studies are needed to document the long-term safety and efficacy of HAART for the prevention of breast-feeding–associated HIV transmission, including among children breast-fed for long periods.

The universal use of HAART in HIV-infected pregnant women irrespective of their clinical or immune status could have several advantages, as shown in resource-rich countries. It avoids using scARV prophylactic regimens, which though effective in reducing MTCT, do not benefit the women's health and are associated with some risk of development of HIV resistance in the mother and infant, particularly with sdNVP [19,30]. However, in low-resource settings, many challenges hinder the universal use of HAART for all pregnant women, including limited access to ARVs, uncertain effects on maternal health from the short-term use of HAART with postpartum interruption, and toxicity concerns about NVP-containing HAART in women with higher CD4 T cell counts [18,31]. It is therefore reassuring that the selective use of HAART by HIV-infected pregnant women with advanced disease, and scARV for PMTCT by pregnant women with less advanced disease yield equivalent results in terms of HIV-free survival in their infants. Furthermore, the use of a ZDV+3TC postpartum “tail” for 7 d reduces the risk of acquiring viral resistance in women receiving scARV for PMTCT including sdNVP [19,32].

We observed a relatively low rate of severe adverse events in HAART-treated HIV-infected pregnant women. Adverse events that did occur were rapidly resolved with appropriate drug management, and unexpected toxicities were absent; together these observations suggest that HAART during pregnancy for women with advanced disease is reasonably safe. Of note, this study did not address the question of the use of NVP-based HAART in women with high CD4 T cell counts. The fact that 7.5% of the women developed toxicities requiring a change of ARV drugs highlights the importance of careful monitoring of pregnant women on HAART. Clinical monitoring can detect many adverse events even without laboratory results: in this study mucocutaneous rashes constituted more than two-thirds of the maternal adverse events, and one woman had severe anaemia with associated clinical symptoms. However, the diagnosis of severe liver toxicity requires frequent laboratory monitoring during pregnancy. Previous reports on adverse events in pregnant women in Mozambique [15], Kenya [33], and Thailand [34] noted that rates of serious hepatic or cutaneous adverse events among pregnant women receiving NVP-containing HAART were comparable to those observed in nonpregnant individuals. Liver toxicity, as indicated by serial liver function tests, was reported in 6.3% of 606 women in the DREAM cohort who were systematically treated by HAART in Mozambique [35]. In our study, we found a rate of 2% of liver toxicity in HAART-treated pregnant women with advanced disease.

We also found that the rate of low birth weight in infants born to mothers treated with HAART was significantly greater than among those exposed to scARV regimens for PMTCT. HAART exposure has been associated with prematurity in some series [16,36] while other studies have found no association [37,38]. Low birth weight might be related to the fact that the mothers had more advanced HIV disease, rather than being a direct effect of HAART itself. Further assessments of these infant outcomes are underway in our cohort [39].

The main limitation of our study is the cohort sample size, with relatively small numbers per treatment group, thus affecting the precision of some MTCT rates and the power to fully perform statistical comparisons between groups. However, we were able to document that the upper confidence limit of the cumulative MTCT rate at 12 mo was less than 7% in the HAART-treated population where breast-feeding was commonly practiced for 5 mo, whereas natural risk may have exceeded 30% [10,11]. Also this study is not a comparative study, but a description of two tiers with one group receiving HAART and another group a short-course of ARVs for PMTCT based on HIV disease staging. Thus, comparisons between the two tiers should be interpreted with caution. A more convincing group for the comparison would be a group of HIV-infected pregnant women eligible for ART who did not receive it. However, it would be unethical to perform such a study in a setting where HAART is available for women who meet eligibility criteria.

Our team has recently summarized available historical data on HIV-infected pregnant women eligible for ART in the same Abidjan population [40]. We estimated the transmission rate at 6 wk at 26.2% in such women having received antepartum a short-course regimen of 4 wk of ZDV alone and practicing predominant breast-feeding. In a comparable group of ART-eligible women who had received 4 wk of ZDV and sdNVP, with short-term breast-feeding, this rate was 10.1%. At 6 mo the corresponding transmission rates were 41.6% and 15.9%, respectively. It is important to note that, in the current study, the infant HIV-free survival rates in the two groups was 11.2% (HAART tier) and 12.1% (scARV tier), not different between infants born from women with high or low risk of HIV transmission. It remains unclear, however, whether the benefits of maternal HAART were counterbalanced by their effect on infants such as early neonatal death in association with prematurity.

This study has several strengths. First, retention was high, with the final HIV status available in more than 86% of live births and only 2% of infants lost to follow-up among HAART-treated mothers. Second, the high level of adherence achieved is a likely explanation for the low rate of MTCT in the HAART-treated tier. Finally, we demonstrated that it is feasible to screen pregnant women for HIV, assess disease status by performing CD4 T cell counts, and quickly initiate HAART. Those who did not require HAART were treated with standard scART regimens for PMTCT, and the vast majority of women and their families were engaged in long-term HIV services. This outcome further demonstrates that PMTCT is an excellent entry point for women and families in HIV care and treatment.

In summary, a two-tiered approach to PMTCT, in which pregnant women with advanced HIV disease receive HAART, and those with less advanced disease receive scARV regimens for PMTCT, was safe during pregnancy and highly effective for the prevention of peripartum and postnatal transmission in an African population. While further investigations in other larger or pooled studies are needed to further explore the balance of benefits and risks related to HAART in breast-feeding mothers, our results provide evidence supporting the most recent WHO guidelines [14]. n

## Supporting Information

Alternative Language Abstract S1 Translation of the Abstract into French by François Dabis (24 KB DOC)

Text S1 IRB Approval Ministry of Health of Côte d'Ivoire: National Ethic Committee consent letter.
(74 KB PDF)

Text S2 Columbia University Medical Centre IRB for the MTCT Plus Initiative (105 KB PDF)

Text S3 Columbia University Human Subjects Protocol Data Sheet (119 KB PDF)

Text S4 IRB Supporting Letter (386 KB PDF)

## Abbreviations

3TC: lamivudine
ART: antiretroviral therapy
ARV: antiretroviral
CI: confidence interval
d4T: stavudine
HAART: highly active antiretroviral treatment
IQR: interquartile range
NVP: nevirapine
PMTCT: prevention of mother-to-child transmission of HIV
sc: short course
sd: single dose
WHO: World Health Organization
VL: viral load
ZDV: zidovudine
