# Supplementary material for: Antiretroviral Treatment and Prevention of Peripartum and Postnatal HIV Transmission in West Africa: Evaluation of a Two-Tiered Approach
Source: PLoS Med. 2007 Aug 21;4(8):e257. doi: 10.1371/journal.pmed.0040257 (PMC1949842; doi:10.1371/journal.pmed.0040257)
Supplement: Supplementary file 1 [file pmed.0040257.sd002.pdf]

MINISTERE DE LA SANTE
ET DE L'HYGIENE PUBLIQUE

REPUBLIQUE DE COTE D'IVOIRE

Union-Discipline-Travail

LE CABINET

4992 J
N° /MSHP/CAB / CNESVS

Abidjan, le 03 NOV. 2006

/-)

Professeur François DABIS
ISPED Université Victor Segalen
Bordeaux (France)
S/C du professeur N'DRI YOMAN T.
UFR des Sciences Médicales
Université d'Abidjan

ABIDJAN

Objet : NL n° 1656/027/MSHP/CNEVS/CAB1/kgo/
du 7 avril 2006 relative à votre demande
d'autorisation à participer à la Collaboration
internationale ART-LINC et KIDS ART LINC.

Professeur,

Le Comité National d'Ethique des Sciences de la Vie et de la Santé a bien reçu votre lettre du 07/06/2006 faisant suite à celle citée en objet.

Après examen de son contenu, de la note d'information aux parents pour la collecte et l'informatisation des données de leur enfant, des accords des sites et des principes de collaboration en date de juin 2006, le Comité National d'Ethique des Sciences de la Vie et de la Santé a noté avec satisfaction la prise en compte de ses observations contenues dans la lettre ci-dessus référencée.

En conséquence, je vous donne mon accord pour la mise en œuvre des Projets de collaboration KIDS-ART-LINC et ART-LINC. Il en est de même de l'utilisation des données des patients décédés et des patients perdus de vue lors de la mise en œuvre des Protocoles ART-LINC ET KIDS ART-LINC.

En vous souhaitant bonne réception, je vous prie d'agréer, Professeur, l'expression de ma considération distinguée.

Pour le Ministre et par Délégation
Le Directeur de Cabinet Adjoint

Dr BLEDI TROUIN Félix
