# Supplementary material for: Antiretroviral Treatment and Prevention of Peripartum and Postnatal HIV Transmission in West Africa: Evaluation of a Two-Tiered Approach
Source: PLoS Med. 2007 Aug 21;4(8):e257. doi: 10.1371/journal.pmed.0040257 (PMC1949842; doi:10.1371/journal.pmed.0040257)
Supplement: Supplementary file 2 [file pmed.0040257.sd003.pdf]

Columbia University Human Subjects Study Description Data Sheet

Protocol: IRB-AAAA5282(Y2M00) Renewal **Protocol Status: Approved** **Effective Date: 01/01/2001**
Expiration Date: 12/31/2007

Originating Department: **MSPH DEAN'S OFFICE (475-00-47500)**
Submitting To: **Medical Center**
Title: **MTCT-Plus Initiative**
Sponsor Protocol Version#:
Abbreviated title: **MTCT Plus Initiaitve**

Affiliated Institutions:
Protocol Begin Date: 00/00/0000
Protocol End Date: 00/00/0000
Previous Protocol Number: 14714

Principal Investigator: Allan Rosenfield (475)

Study Description

Previously Paper Protocol 14714

Columbia University IRB
Approved for use until: 12/31/2007
Superseded
