# Supplementary material for: Antiretroviral Treatment and Prevention of Peripartum and Postnatal HIV Transmission in West Africa: Evaluation of a Two-Tiered Approach
Source: PLoS Med. 2007 Aug 21;4(8):e257. doi: 10.1371/journal.pmed.0040257 (PMC1949842; doi:10.1371/journal.pmed.0040257)
Supplement: Supplementary file 3 [file pmed.0040257.sd004.pdf]

Columbia University Human Subjects Protocol Data Sheet

Protocol: IRB-AAAA5282(Y2M00) Renewal | **Protocol Status: Approved** | **Effective Date: 01/01/2001**
Expiration Date: 12/31/2007

Originating Department: **MSPH DEAN'S OFFICE (475-00-47500)**
Submitting To: **Medical Center**
Title: **MTCT-Plus Initiative**
Sponsor Protocol Version#:
Abbreviated title: **MTCT Plus Initiaitve**

Affiliated Institutions:
Protocol Begin Date: 00/00/0000
Protocol End Date: 00/00/0000
Previous Protocol Number: 14714

Personnel Staff

Name/Phone	Role	COI-Date	Courses	Dept	Edit/ View	Procedure Experience
Allan Rosenfield, (ar32) 212-305-3929 Professor	Principal Investigator	09/26/2006	1. HS GCP - Epid. & Soc. Behav. - 09/28/2000<br>2. HIPAA - 03/22/2005	475	Edit	
Jessica Arrighi, (jaw83) (212) 342-0535 Admin Coord	Initiator	08/09/2004		478	Edit	
*Wafaa El-sadr, (wme1) 212 939 2936 Prof Clin/ Assoc Attend	Co-Investigator	11/07/2006	1. HS GCP - Patient/Clinical - 03/19/2001<br>2. HIPAA - 01/02/2004	527	Edit	

Renewal

Subject Enrollment Status:
Additional relevant information if needed to further explain Study Status:
Date enrollment began at our site: 00/00/0000
Summary of any recent literature or interim findings:
Changes in the literature since the last renewal application:
List of papers pending or published:
Synopsis of the results to date:
If this renewal includes a modification:
Certificate of Confidentiality (COC) obtained:
If COC obtained, expiration date of Certificate: 00/00/0000

Subjects

Columbia University IRB
Approved for use until: 12/31/2007
Superseded

Is this a multicenter study?	
Target accrual at all sites:	0
Number of Subjects accrued to date at all sites:	0
Target accrual at this site:	0
Number accrued to date at this site:	0
Number completed at this site:	0
Number still on study at this site:	0
Number who withdrew of own initiative(including those lost to follow-up):	0
Number removed by researcher:	0
Number of subjects accrued since the last IRB approval:	0
Number anticipated to accrue next approval period:	0
Number of subject complaints at this site:	0

Explain subject complaints:

Explain subjects removed by researcher:

Explain subjects who withdrew of own initiative:

Explain incomplete accrual at our site:

Explain problems meeting anticipated accrual number:

Strategies to remedy these problems:

Have you enrolled subjects using the Short Form Consent Process for Non-English speaking subjects?:
Please indicate the number of subjects enrolled for each language:

Subject justification: Previously Paper Protocol 14714

Subject compensation: Previously Paper Protocol 14714

Compensation justification: Previously Paper Protocol 14714

Consent form waiver/alteration request: Previously Paper Protocol 14714

Recruitment url:

Columbia University IRB
Approved for use until: 12/31/2007
Superseded

Oversight Monitoring/Renewal
Adverse Event

Summary of all Adverse Events to date at all sites:

Number of deaths at this site: 0

Has your assessment of risks and benefits changed since the last submission?:

If Yes, describe assessment of risks and benefits:

If there is a Data Safety Monitoring Committee for this study, is the most recent report attached?:

Explanation why the most recent safety monitor report is not attached:

If this is a federally supported or multi-center study, is the most recent progress report of this study attached?:

Explanation why the most recent report is not attached:

Research

Research procedures:

Research facilities:

Scientific abstract: Previously Paper Protocol 14714

Lay abstract: Previously Paper Protocol 14714

Approval Personnel

Electronic Signature: Allan Rosenfield (475) - Principal Investigator ***Date: 00/00/0000 NOT YET SIGNED***
Electronic Signature: Wafaa El-sadr (527) - Co-Investigator ***Date: 00/00/0000 NOT YET SIGNED***

Columbia University IRB
Approved for use until: 12/31/2007
Superseded
