# Supplementary material for: Antiretroviral Treatment and Prevention of Peripartum and Postnatal HIV Transmission in West Africa: Evaluation of a Two-Tiered Approach
Source: PLoS Med. 2007 Aug 21;4(8):e257. doi: 10.1371/journal.pmed.0040257 (PMC1949842; doi:10.1371/journal.pmed.0040257)
Supplement: Supplementary file 4 [file pmed.0040257.sd005.pdf]

COLUMBIA UNIVERSITY
MEDICAL CENTER

Institutional Review Board
(CUMC IRB)
722 W. 168th Street 4th Flr.
New York, NY 10032
212.305.5883 Tel
212.305.1316 Fax

www.cumc.columbia.edu

May 9, 2006

Wafaa El-Sadr, MD
Department of Medicine
Harlem Hospital Center
MLKB 3

**Re:**	**IRB Determination**	
**Protocol #:**	**AAAA5282**	**Award #: MTCTP CU51676001**
**Title:**	**MTCT-Plus Initiative**	

Dear Dr. El-Sadr:

On April 1, 2006, this protocol was reviewed by the IRB Chair, and a determination was made that it is "Not Human Subjects Research per 45 CFR 46". The protocol, while it is research, does not involve human subjects as defined by 45 CFR 46, i.e., there is no interaction with subjects, there is no intervention, and private, identifiable information is not being collected. Thus, further IRB review is not needed.

Although this study as described does not require review by the IRB, all research activities that involve humans or specimens from humans must be submitted to the IRB for a determination of whether IRB review is required.

Research means a systematic investigation, including research development, testing and evaluation, designed to develop or contribute to generalizable knowledge. Activities which meet this definition constitute research for purposes of this policy, whether or not they are conducted or supported under a program which is considered research for other purposes. For example, some demonstration and service programs may include research activities.

Human subject means a living individual about whom an investigator (whether professional or student) conducting [research obtains (1) data through intervention or interaction with the individual, or (2) identifiable private information].

If you have any questions regarding this approval, please contact the IRB office at (212) 305-5883. Columbia University appreciates your commitment towards the ethical conduct of human research.

Sincerely,

Lauren Privitera, CIP
Team #2 Manager
Columbia University Medical Center IRB
IRB/taw
