# Supplementary material for: Antiretroviral Treatment and Prevention of Peripartum and Postnatal HIV Transmission in West Africa: Evaluation of a Two-Tiered Approach
Source: PLoS Med. 2007 Aug 21;4(8):e257. doi: 10.1371/journal.pmed.0040257 (PMC1949842; doi:10.1371/journal.pmed.0040257)
Supplement: Supplementary file 5 [file pmed.0040257.sd001.doc]

**Abstract in french**

**Background:** Le traitement antirétroviral hautement efficace (HAART) a récemment été recommandé pour les femmes enceintes ayant besoin de traitement pour elles-mêmes dans les pays à ressources limités.

**Objectif:** Nous avons évalué l’efficacité à court terme (4 semaines) et à long terme (12 mois) d’une stratégie de prévention de la transmission de la mère à l’enfant (PTME) du VIH en Afrique, qui a pour but de traiter par une HAART les femmes enceintes ayant les critères d’éligibilité OMS et de donner des régimes prophylactiques courts de médicaments antirétroviraux pour les femmes atteint d’une maladie VIH moins avancée.

**Méthodes:** L’Initiative MTCT-Plus est un programme de soins et de traitement centré sur la famille enrôlant dans des multiples pays des femmes enceintes et en post-partum et leurs familles. Des femmes enceintes ont été enrôlées à Abidjan, Côte d’Ivoire, recevant soit un traitement ARV hautement efficace pour elle-même soit un régime prophylactique court selon leurs critères cliniques et immunologiques. La charge virale ARN-VIH a été mesurée pour le diagnostic de l’infection peri-partum à 4 semaines et le statut VIH final a été documenté par un test sérologique rapide à ≥12 mois ou plus tôt par une PCR. La méthode de Kaplan-Meier a été utilisée pour évaluer le risque de transmission du VIH de la mère à l’enfant et la survie des enfants sans VIH

**Résultats**: Entre août 2003 et juin 2005, 107 femmes ont débuté une HAART en médiane à 30 semaines de gestation, 102 avec une combinaison de zidovudine (ZDV), lamivudine (3TC) et nevirapine (NVP) poursuivie en post-partum ; les autres 143 femmes enrôlées ont reçu un régime court de PTME, dont 103 une combinaison de ZDV+3TC pendant 2 mois associé à une dose unique de NVP pendant le travail; 75% des nouveaux-nés ont été allaité au sein pendant 5 mois en moyenne. Dans les deux groupes confondus, le taux de transmission peri-partum était de 2.2% (intervalle de confiance 95% [CI] 0.3-4.2%) et le taux cumulatif à 12 mois était de 5.7% (IC 2.5-9.0). La probabilité pour un enfant de décéder ou d’acquérir une infection VIH était de 4.3% (IC 1.7-7.0 à 4 semaines et 11.7% (IC 7.5-15.9) à 12 mois.

**Conclusion**: Cette stratégie semble être sûre et hautement efficace pour la PTME à court et à long terme dans les pays à ressources limitées. Cette constatation pousse à améliorer l’accès à une HAART pour les femmes enceintes ayant besoin de traitement pour elles-mêmes dans les pays à ressources limitées comme le recommande explicitement l’OMS.
